# Improving Health Outcomes Through Treatment Sequencing Optimization in Multiple Myeloma: A Simulation Model in Transplant‐Ineligible Patients

**DOI:** 10.1002/cnr2.70027

**Published:** 2024-10-07

**Authors:** C. Geraldes, M. Neves, R. Bergantim, C. Silva, F. Leal da Costa

**Affiliations:** ^1^ Centro Hospitalar Universitário de Coimbra Coimbra Portugal; ^2^ Fundação Champalimaud Lisboa Portugal; ^3^ Faculty of Medicine of the University of Porto (FMUP), Porto, Portugal; i3S ‐ Institute for Research and Innovation in Health, University of Porto, Porto, Portugal; Cancer Drug Resistance Group, Institute of Molecular Pathology and Immunology of the University of Porto (IPATIMUP), Porto, Portugal; Department of Hematology Centro Hospitalar Universitário de São João Porto Portugal; ^4^ Institute for Evidence‐Based Health (ISBE) Lisboa Portugal; ^5^ Instituto Português de Oncologia Lisboa Portugal

**Keywords:** multiple myeloma, optimization, outcomes, overall survival, simulation, treatment sequencing

## Abstract

**Objectives:**

Patients with multiple myeloma often require multiple treatment lines. The order in which treatments are sequenced has impact on clinical outcomes. This study aimed to estimate progression‐free survival (PFS) and overall survival (OS) with common treatment sequences used in Portugal and the incremental benefit of an optimal sequence in transplant‐ineligible patients with multiple myeloma.

**Methods:**

A state‐transition sequential model with a five‐health state conceptual structure was developed to simulate and compare survival outcomes between treatment sequences up to four lines of treatments. Data sources included randomized clinical trials and indirect treatment comparisons. A panel of Portuguese hematologists listed four most common treatment sequences and optimal sequence of choice in transplant‐ineligible patients.

**Results:**

Our simulation estimated an OS between 6.1 and 7.8 years using the most common sequences, with VMP + DRd + Pd + Kd as the most effective (7.8 years). Optimal sequence of choice (DRd + PVd + Kd + Vd) achieved OS of 9.8 years and may extend OS in 2.0–3.7 years vs. most common sequences (26%–61% increase). This benefit was mostly explained by extended PFS in the first line of treatment.

**Conclusion:**

Model results demonstrate that choosing the most effective treatment upfront is crucial in delaying disease progression thus yielding better survival outcomes in transplant‐ineligible patients. There was a clear survival benefit in using daratumumab‐based regimens in first line. This modelling exercise highlights the need to raise awareness around the impact of sequencing strategies to improve patient's outcomes.

## 1 Introduction

Multiple myeloma (MM) is an incurable and high burden [1] disease characterized by the proliferation of malignant plasma cells within the bone marrow causing increased susceptibility to infections and a wide range of symptoms.

In 2020, approximately 176 404 people worldwide were diagnosed with MM accounting for 0.9% of all cancer diagnoses. The MM global crude incidence was 2.3/100 000 people worldwide, 6.8/100 000 people in Europe and 8.7/10 000 in Portugal [2]. Over the past decade, MM incidence has risen by 126% globally [3].

The primary goal of MM treatment is to delay progression and prolong overall survival (OS) [4]. Treatment landscape has radically changed over the past decade with OS almost doubling with novel multi‐drug combination regimens [5]. The availability of new effective treatment options in newly diagnosed MM patients requires a definition of optimal treatment sequencing.

Patients typically start treatment with a combination of drugs across various pharmacotherapeutic groups, with or without autologous stem cell transplantation (ASCT). However, patients invariably relapse and will often require multiple lines of treatment. The order in which treatments are sequenced differs in clinical practice by multiple reasons and has a recognized impact on patients' clinical outcomes [6].

Optimizing treatment sequencing is critical in achieving the best long‐term outcomes in clinical practice [6]. Using the most effective treatment upfront is crucial in delaying disease progression, hence yielding better survival outcomes for patients [6]. In general, the first remission is more durable than subsequent remissions and is associated with a deeper response.

There is a lack of comparative clinical trials and consensus on the optimal sequence to guide practitioners [7]. Ideally, real‐world sequencing data would inform the efficacy of all treatment sequences. However, evidence generation becomes challenging due to the growing number of available MM therapies and the significant number of potential sequences that emerge when a patient may undergo multiple lines of treatment [5, 8, 9].

Towards this evidence gap, the Local Use of Myeloma Optimized Sequences (LUMOS) model was developed to simulate the impact of using alternative treatment sequences on patient's health outcomes [10].

The objective of the current analysis was to estimate and compare OS and progression‐free survival (PFS) of the treatment sequences most used in clinical practice in MM transplant ineligible (TIE) patients in Portugal and estimate incremental health gains vs. an optimal treatment sequence using LUMOS model.

## 2 Methods

### 2.1 Model Structure

**FIGURE 1:**
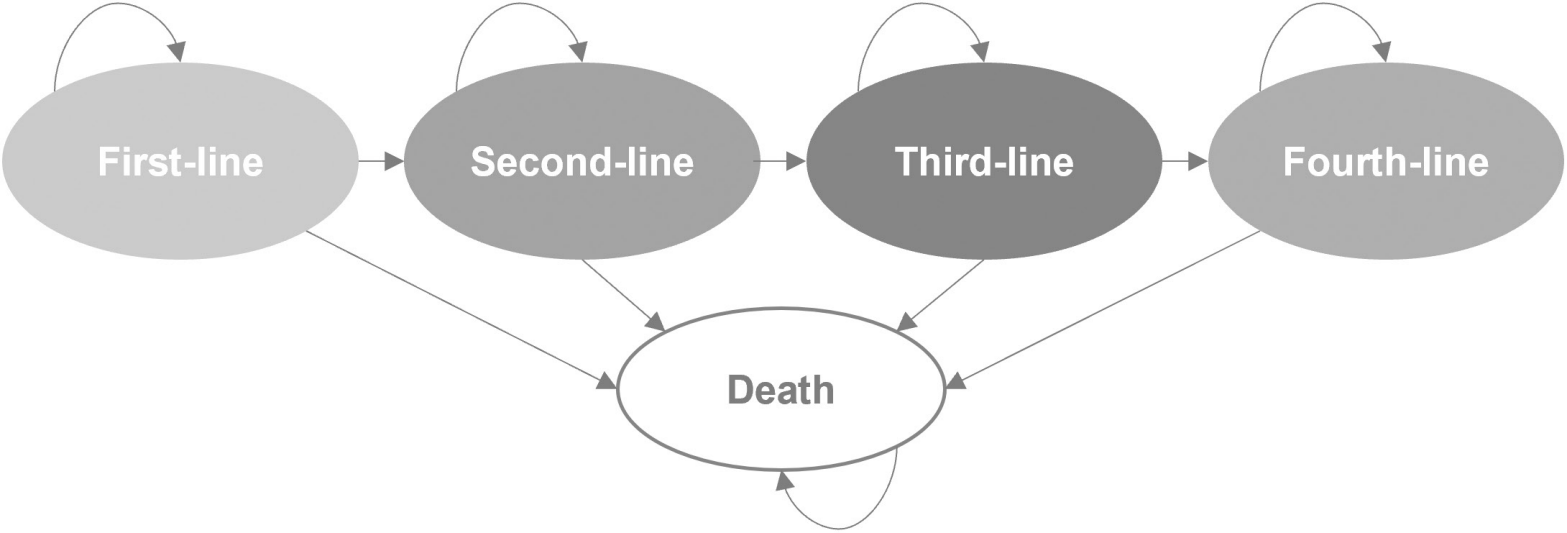
Estimates of PFS and OS by treatment line by treatment sequence. D, daratumumab; DRd, daratumumab + lenalidomide + dexamethasone; Kd, carfilzomib + dexamethasone; OS, overall survival; Pd, pomalidomide + dexamethasone; PVd, pomalidomide + bortezomib + dexamethasone; VCd, bortezomib + cyclophosphamide + dexamethasone; Vd, bortezomib + dexamethasone; VMP, bortezomib + melphalan + prednisone; VRd, bortezomib + lenalidomide + dexamethasone.

LUMOS model is a health state‐transition treatment sequencing survival model developed to simulate and compare survival outcomes from multiple treatment sequences in MM (Figure 1), detailed elsewhere [10]. Newly diagnosed patients start a first line treatment administered until disease progression. At first relapse, the patient may transition to a subsequent treatment line, and so forth up to a maximum of four treatment lines or move directly until death (absorbing state). In the Portuguese model simulation, once progression occurred after the fourth line of therapy, assumption was made that patient had terminal disease and was considered as transitioning to death. The model was developed considering a 2‐week cycle length providing sufficient granularity to track outcomes accurately and ensuring an adequate computational time. Half‐cycle correction was not applied due to the short cycle length. A lifetime time horizon (40 years) was used to capture all relevant benefits of the different treatment sequences. Survival clinical outcomes were not discounted given the objective of the analysis. LUMOS model was validated as described in Supporting Information.

### 2.2 Population

The current simulation was performed in the newly MM diagnosed TIE patient setting.

### 2.3 Clinical Data

Overall, LUMOS model was informed for the newly‐diagnosed, relapse or refractory, and double‐refractory setting using data from clinical trials (Table S1), network meta‐analyses (NMA) or matched‐adjusted indirect comparisons (MAIC). For the first three lines of treatment, transition probabilities between health states were informed by PFS curves by treatment line. Fourth line modelling followed a standard partitioned survival approach using PFS and OS curves to estimate the proportion of patients who were progression‐free, post‐progression or dead.

In the first line setting, patient‐level data from MAIA [11] and ALCYONE [12] trials, performed in the TIE setting, were used to inform PFS curves for daratumumab‐based regimens and direct comparators of each study (daratumumab, lenalidomide and dexamethasone [DRd] and lenalidomide and dexamethasone [Rd] in MAIA; daratumumab, bortezomib, melphalan, and prednisone [DVMP] and bortezomib, melphalan and prednisone [VMP] in ALCYONE) with a median follow‐up of 56.2 and 40.1 months, respectively. Hazard ratios from a NMA of frontline MM treatments [13, 14] (Figure S1) were applied lolo the Rd curve (reference curve) to obtain PFS curves for the other first‐line regimens. A parametric curve was fitted to each PFS curve following the recommendations from the National Institute for Health and Care Excellence Technical Support Document [15] and Portuguese Economic Evaluation Guidelines [16]. Curve selection was guided through a combination of visual and statistical fit, and clinical plausibility of the long‐term extrapolations. The sub‐distribution of PFS events (progression or death) was informed by real‐world evidence data from Fonseca et al. [17].

For the relapsed and refractory setting, since licensed regimens may be used at second line, third line and fourth line, patient‐level data from CASTOR [18] and POLLUX [19] trials were used to estimate line‐specific PFS and OS curves for daratumumab‐based regimens and direct comparators (daratumumab, bortezomib and dexamethasone [DVd] and bortezomib and dexamethasone [Vd] from CASTOR; DRd and Rd from POLLUX), with a median follow‐up of 50.2 and 54.8 months, respectively. There was no unique NMA for all the regimens available in this setting. Therefore, hazard ratios were obtained from a NMA anchored in Rd [20] (Figure S2) and a NMA anchored in Vd [21] (Figure S3). These hazard ratios were then applied to PFS reference curves to obtain the curves for the other regimens. The sub‐distribution of PFS events followed the same method as in first line.

For the double‐refractory setting (from the third line onwards), although regimens in the relapsed and refractory setting may be used, some treatments are exclusively licensed in this setting. As such, PFS and OS reference curves were estimated for daratumumab monotherapy using pooled patient‐level data from SIRIUS [22] and GEN501 [23] trials according with the number of previous treatment lines. PFS and OS curves of other treatment regimens, available for this setting, were estimated using hazard ratios from an existing MAIC [24]. OS and PFS reference curves for Pd were derived through digitization, patient‐level data recreated and extrapolation of OS and PFS data from MM‐003 [25], ELOQUENT‐3 [26], and ICARIA‐MM [27] trials.

For the first three lines of treatment, the distribution of PFS events between patients who progress to the next treatment line or progress to death (attrition rates) were informed by line‐specific real‐world data published for the TIE population [17].

### 2.4 Treatment Regimens

Treatment regimens for MM TIE patients licensed by the European Medicines Agency and available in Portugal as of September 2022 were selected in LUMOS model for each treatment line setting (Table 1).

**TABLE 1:** Treatment regimens in Portugal for MM TIE patients (September 2022).

Newly diagnosed	Relapsed or refractory	Double refractory
DRd	DRd	D
DVMP	DVd	Pd
Rd	Rd	IsaPd
VRd	Vd	
VMP	VCd^a^	
	KRd	
	Kd	
	IRd	
	PVd	
	EloRd	

Abbreviations: C, cyclophosphamide; D, daratumumab; d, dexamethasone; DVMP, daratumumab + bortezomib + melphalan + prednisolone; Elo, elotuzumab; I, ixazomib; Isa, isatuximab; K, carfilzomib; M, melphalan; P, pomalidomide; R, lenalidomide; V, bortezomib; VMP, bortezomib + melphalan + prednisolone.

^a^ Not approved.

### 2.5 Definition of National Clinical Practice

An explanatory sequential mixed‐methods design was adopted to define the clinical practice regarding treatment sequencing in Portugal. Four Portuguese haematology experts from four main Portuguese hospitals in MM patient management and treatment were invited and accepted to participate. In the first phase, an online survey was sent to clinical experts to collect quantitative data (their best estimate/opinion using a statistical measure like a mean or percentage) for characterization of both MM patient population and most common treatment sequences. For the second phase, qualitative data collection was performed through a virtual meeting conducted in September 2022. Aggregated data from the quantitative survey was presented and discussed between the experts to achieve agreement on the most common treatment sequences used in clinical practice and the optimal treatment sequence of choice available in Portugal in MM TIE patients at the time the expert panel was held. Treatment sequences were inputted in the LUMOS model and a live online simulation was performed, with model outcomes discussed and validated by clinical experts. A trained moderator followed a semi‐structured script with open‐ended questions to drive ongoing discussions, supported by a minute taker.

### 2.6 Model Outcomes

Estimated outcomes included average PFS, defined as time spent free of progression in each line of treatment per sequence, and OS per treatment sequence. For the most common treatment sequences, considering limitations in available local clinical practice data to inform the weight of each sequence among the overall most common sequences defined by clinical experts, a non‐weighted OS average is presented.

### 2.7 Projection of Optimal Sequence Impact

To quantify the potential impact of treating new MM TIE patients per year in Portugal with the optimal sequence of choice, based on national epidemiologic data and LUMOS simulation outcomes, a simplified calculation was performed to estimate the number of life years (LY) that could be saved if all new yearly TIE patients were treated with the optimal sequence in comparison with the four most common sequences used in current clinical practice.

## 3 Results

### 3.1 Selected Sequences

Upon agreement, clinical experts identified a total of four most common treatment sequences in their clinical practice (all locally reimbursed, except for VCd) and one optimal treatment sequence (Table 2) for MM TIE patients as of September 2022.

**TABLE 2:** Most common treatment sequences and optimal sequence for TIE MM patients.

Sequences	First line	Second line	Third line	Fourth line
Most common sequences	VMP	DRd	Pd	Kd
	VMP	DRd	Kd	Pd
	Rd	VCd	D	Pd
	Rd	VCd	Pd	D
Optimal sequence	DRd	PVd	Kd	Vd

Abbreviations: C, cyclophosphamide; D, daratumumab; d, dexamethasone; K, carfilzomib; M, melphalan; P, pomalidomide; R, lenalidomide; V, bortezomib; VMP, bortezomib + melphalan + prednisolone; first line, first‐line, second line, second‐line, third line, third‐line, fourth line, fourth‐line.

### 3.2 Overall Survival

**FIGURE 2:**
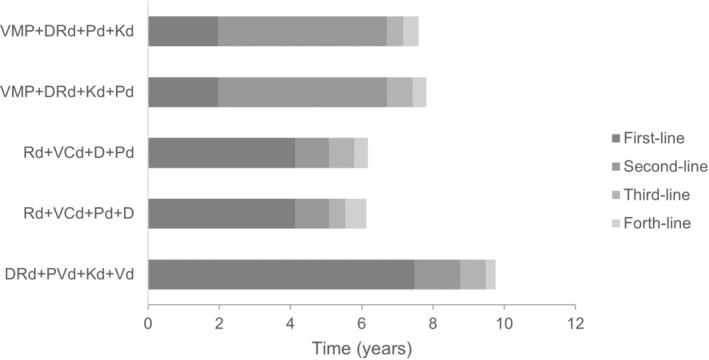
Model structure.

A comparison of all sequences in terms of OS is presented in Figure 2. Our simulation estimated an OS for TIE patients ranging between 6.1 and 7.8 years for most common sequences used in clinical practice, with a non‐weighted average of 6.9 years. VMP + DRd + Pd + Kd and VMP + DRd + Kd + Pd standed as the most effective in terms of OS, with 7.8 and 7.6 years, respectively, among the four most common sequences in clinical practice.

The optimal sequence by physicians' choice, DRd + PVd + Kd + Vd, achieved an OS estimate of 9.8 years which represents a survival benefit vs. the most common sequences ranging between 2.0 and 3.7 years, corresponding to a survival increase of 26%–61%, respectively (Table 3).

**TABLE 3:** OS incremental analysis versus optimal sequence in MM TIE patients.

		PFS				OS	∆OS vs. optimal sequence
		First line	Second line	Third line	Fourth line		
Most common sequences	VMP + DRd + Pd + Kd	1.97	4.74	0.46	0.42	7.59	−2.17
	VMP + DRd + Kd + Pd	1.97	4.74	0.72	0.38	7.81	−1.95
	Rd + VCd + D + Pd	4.13	0.95	0.71	0.38	6.17	−3.59
	Rd + VCd + Pd + D	4.13	0.95	0.46	0.59	6.13	−3.63
Optimal sequence	DRd + PVd + Kd + Vd	7.48	1.29	0.71	0.28	9.76	—

Abbreviations: C, cyclophosphamide; D, daratumumab; d, dexamethasone; K, carfilzomib; M, melphalan; P, pomalidomide; R, lenalidomide; V, bortezomib; VMP, bortezomib + melphalan + prednisolone; ∆, absolute difference in OS between most common sequences versus optimal sequence.

### 3.3 Progression‐Free Survival

Regarding the four most common treatment sequences used in national clinical practice, time spent free of progression was highest in first or second line, markedly decreasing in third or fourth line. According to model simulation, PFS averaged between 1.97 and 4.13 years in first line, 0.95 and 4.74 years in second line, 0.46 and 0.72 years in third line, and 0.38 and 0.59 years in fourth line (Table 3 and Figure 2).

First‐line PFS is notoriously increased with optimal sequence vs. the most common current sequences, namely 3.8 and 1.8‐fold when compared with VMP + DRd + Pd + Kd or VMP + DRd + Kd + Pd and Rd. + VCd + D + Pd or Rd. + VCd + Pd + D sequences, respectively (Table 3).

### 3.4 Projection of Optimal Sequence of Choice in Patient Life Years

Considering a total of 886 MM new cases in Portugal per year [2] of whom 60% are considered TIE and 15% of those will achieve a fourth line of treatment, we estimate that a total of 338 LY can be gained using the optimal regimen of choice when compared with the non‐weighted average of the most common treatment sequences (Table 4).

**TABLE 4:** Projection of life years saved with optimal sequence versus most common regimens in MM TIE patients in Portugal.

	Estimate	Source
MM new cases per year	886	GLOBOCAN [2]
Ineligible for transplant	532	Expert panel (60%)
Achieve a fourth line	93	Expert panel (17.5%)
LY if new diagnoses are treated with four most common regimens (non‐weighted OS mean)	570	Calculation
LY if new diagnoses are treated with optimal sequence	908	Calculation
LY gained	338	Calculation

Abbreviations: LY, life years; OS, overall survival.

## 4 Discussion

A targeted non‐systematic literature search performed using Pubmed (search terms sequence AND cancer AND treatment) showed that published articles on treatment sequencing in Oncology increased exponentially between 2009 and 2020, but only about half of the studies compared different treatment sequences. Most articles focused on solid tumors (lung, breast, and colorectal cancer), with treatment sequences rarely compared beyond second line. No Portuguese publications were found.

LUMOS simulation model was developed to address the scarcity of sequencing studies in MM [28], to leverage evidence‐based treatment decisions and support tailoring of therapeutic strategies in real‐world practice.

Using expert clinical experience in MM management, we were able to adapt LUMOS model to the national clinical practice, with the inclusion of the four most common treatment sequences and an optimal chosen sequence (DRd + PVd + Kd + Vd) enabling the comparison of OS between different sequences. To our knowledge, this is the first simulation model used to compare clinical outcomes across treatment sequences with four treatment lines in MM in Portugal.

We focused on TIE subpopulation since 60% of MM new cases are not eligible for transplant in Portugal, as agreed between panel experts. Moreover, these patients have a higher unmet need and a higher attrition rate compared with TE subpopulation.

According to experts' opinion, about 15%–20% of TIE patients are expected to reach a fourth line of treatment which is aligned with the estimates provided by a large European real‐world MM study [29]. Nevertheless, experts foresee that the number of patients achieving more advanced treatment lines will raise due to uptake of innovative MM treatments in clinical practice.

LUMOS model demonstrated that higher efficacy and time on treatment in early lines are the major drivers for optimizing long‐term outcomes in MM in TIE patients. Therefore, using the most effective treatment upfront is crucial as these will be the most impactful on long‐term clinical outcomes, delaying disease progression and extending OS.

The best outcome was achieved with the use of daratumumab‐based in first line (optimal chosen sequence). This result is in accordance with results of a published meta‐analysis that showed that daratumumab‐based regimens improve efficacy and increase survival of patients with MM [30].

The Portuguese expert panel defined the optimal sequence based on: (a) efficacy of first‐line daratumumab‐based regimens in OS extension and its good tolerability profile; (b) and for second line the most effective and adequate regimens in a post‐daratumumab setting among authorized regimens, including PVd approved by EMA in second line. Furthermore, the optimal sequence is aligned with recently published Portuguese guidelines [31]. It should be highlighted that at the time the panel was held, bispecific antibodies and CAR T therapies were not being used in Portuguese clinical practice.

According to our model estimates, the use of this optimal sequence would represent a survival gain of 2.0–3.7 years when compared with the most common sequences in current national clinical practice. This gain is mostly explained by the incremental PFS in first line.

Clinical experts listed advantages in adopting the optimal sequence, including gains in both PFS and OS, and the previous positive clinical experience with daratumumab‐based regimens in the relapsed setting, particularly second line their experience with daratumumab in second line bringing confidence for its use in first line. Challenges were also identified, including risk of infection in first line (which decreases over time with clinical experience), continuous therapy (vs. fixed‐duration regimens such as VMP), and hospital budget concerns.

In 2014, Heeg et al. [32] designed a first early analytical framework comparing 17 sequences in TIE patients but results are out of date due to profound differences in the therapeutic landscape.

In 2013, Baz et al. [33] retrospectively reviewed data from patients with MM to compare two sequences (lenalidomide‐bortezomib vs. bortezomib‐lenalidomide and found that the use of bortezomib in first line was associated with improved survival but only for patients with baseline renal insufficiency.

More recently, Blommestein et al. [34] performed a cost‐effectiveness analysis in MM TIE including 30 novel treatment sequences up to a maximum of third line. The largest estimated OS was 7.5 years (with DVMP in first line and KRd or EloRd as second line, or VMPT‐VT in first line and DRd as second line) which is aligned with the best estimate found among our most common treatment sequences (OS of 7.4 years with VMP + DRd + Kd + Pd, considering a cut‐off at third line). The authors also found an average OS of 5.7 years for the VMP + DRd + Pd and of 5.8 years for the VMP + DRd + Kd which are 1.5 years lower than LUMOS OS estimates in sequences VMP + DRd + Pd + Kd and VMP + DRd + Kd + Pd (considering a cut‐off at third line). Nevertheless, Blommestein et al. [34] and LUMOS models are based on different assumptions, data sources, and treatment sequences which limit comparisons and differences in results.

In 2021, Petrucci et al. [35] presented the LUMOS model customization for the Italian setting. Compared sequences were different but overall conclusions were aligned with our findings.

A state‐transition modelling approach was used for its advantage of being relatively simple to implement using accessible software (Microsoft Excel) and widely used within oncology field, particular at earlier stages of disease where subsequent treatment lines are explicitly modelled.

Transition probabilities between treatment lines were estimated by PFS curves. Time to next treatment data would be ideal, but since these endpoints are rarely and/or not consistently reported there would be limitations in generating comparative evidence. Thus, PFS was used instead being assumed that a treatment is administered until progression of the disease (or maximal therapy duration) and that a new treatment line is initiated once the patient progress. LUMOS model estimated PFS and OS which were selected for being relevant hard endpoints in MM and reflect the outcomes measured under clinical development programs.

In our simulation the treatment pathway in TIE MM was simulated until fourth line. Studies have shown that only a small proportion of patients receives a 5 L therapy (1% according to Yong et al. [29]). Simulated OS may be underestimated since it did not account for post‐progression survival after a fourth line relapse, although expert opinion indicated a residual impact in model results since survival after relapse without further treatment is commonly residual.

The attrition rates considered in the model were considered constant within each treatment line independently of treatment sequence which may not match attrition rates seen in real‐world practice. Nevertheless, according to a recent Portuguese study [36] using secondary data from 11 NHS Portuguese hospitals, drop‐out rate varied between 28% in first line and 19% in third line to fourth line.

Characteristics of TIE MM patients are heterogeneous and therefore there is no unique ideal sequence for all patients [5]. Indeed, treatment should be tailored according to patients' clinical characteristics (age, frailty, and comorbidities).

In the future, LUMOS may be leveraged to explore the impact on health outcomes in specific patient subgroups allowing to understand the impact of treatment sequence optimization in specific patient profiles.

Methodology used in LUMOS to estimate survival outcomes has been used in previous simulations [34, 37, 38] as an exploratory alternative to overcome the impossibility of performing clinical studies for all possible MM treatment sequencies. Also, there may be discrepancies between clinical trial and real‐world outcomes, but overall, the experts agreed with the estimates provided by the model mentioning to be aligned with what they would expect in their clinical practice.

The panel acknowledged that treatment sequencing evidence is important due to its impact on patient outcomes and therefore it should be taken into consideration in clinical practice. Treatment sequencing standardization in national clinical practice may be hard to achieve due to patient clinical heterogeneity (e.g., differences in patient population characteristics), hospital clinical practices (e.g., differences in hospital treatment protocols, patient access disparities) or/and institutional/regulatory reasons (e.g., lack of alignment between recommended vs. reimbursed drugs). Efforts should be taken for a public discussion around sequencing in national clinical practice. National guidelines were published in 2023 contributing as the first step for this standardization [39]. Most experts follow European guidelines, but it was recognized that there should be periodic updates to include latest innovations.

## 5 Conclusions

The rapidly evolving treatment landscape in MM allows a vast number of possible treatment sequences and adds complexity in selecting the most appropriate sequence to optimize patient outcomes.

LUMOS is an evidence‐based support tool that can be adapted to country‐specificities and continually updated with forthcoming MM treatments to support treatment‐sequencing decision. Conduction of clinical trials to compare such a high number of treatment sequences is not feasible and therefore simulation models like LUMOS should be used as general guiding tools to support clinicians in optimizing MM patient treatment.

Our analysis demonstrates the importance of selecting the most‐effective treatments upfront, to ensure optimal patient outcomes. Time spent in progression free state decreases with each subsequent line of treatment, and the magnitude of the effect seen in the third line and fourth line is not as significant as in earlier treatment lines. Daratumumab‐based regimens in first line demonstrated the longest PFS and clinically significant OS extension vs. current common treatment sequences in national clinical practice.

In the future, with evidence accumulation from clinical trials and real‐world practice, the conduction of big data studies together with the use of machine learning methods can be used to establish optimal treatment sequences based on individual patient characteristics. Meanwhile, this modelling exercise highlights the need to raise awareness around the need to explore sequencing strategies to optimize MM long‐term outcomes in clinical practice and helpfully contribute to an informed treatment decision‐making.

## Author Contributions

**C. Geraldes:** writing – review and editing, formal analysis, validation, conceptualization, methodology, writing – original draft. **M. Neves:** writing – review and editing, formal analysis, validation, conceptualization, methodology, writing – original draft. **R. Bergantim:** writing – review and editing, formal analysis, validation, conceptualization, methodology, writing – original draft. **C. Silva:** writing – original draft, writing – review and editing, validation, formal analysis, methodology, conceptualization. **F. Leal da Costa:** writing – review and editing, conceptualization, formal analysis, methodology, writing – original draft, validation.

## Disclosure

Under this Open Access license, the authors agree that anyone can reuse this article in whole or part for any purpose, for free, but not for commercial purposes. Anyone may copy, distribute, or reuse the content as long as the author and original source are properly cited.

## Ethics Statement

This simulation only collected expert opinion (estimates using statistical measures like mean or percentage, not real data) for LUMOS customization which does not fulfil criteria for IRB/EC submission, and therefore this analysis was considered exempted from IRB approval (Portuguese Law no. 58/2019, 8 of August; https://diariodarepublica.pt/dr/detalhe/lei/58‐2019‐123815982).This simulation was considered exempt as per national guidelines. Therefore, ethics approval from IRB/EC was not obtained.

## Conflicts of Interest

C.G. received fees for scientific meetings and advisory boards from Celgene, BMS, Janssen, Amgen, Pfizer, Takeda, Sanofi, or Gilead; R.B. received fees for consultancy, research funding, or speaker from Amgen, Janssen, BMS, or Takeda; F.L.d.C. received fees for Consultancy or Research from BMS/Celgene, Janssen, Takeda, Amgen, Pharmamar, Servier, Astellas, Pfizer, Viforpharma, 2Logical, or Sanofi; M.N. received fees for consultancy or speaker from Amgen, Janssen, BMS, Sanofi, Pfizer or Takeda. C.S. is employed by Institute for Evidence‐Based Health contracted by Janssen‐Cilag Portugal to assist with medical writing in the development of this manuscript.

## Supporting information


Figure S1.

Figure S2.

Figure S3.

**Data S1.** Supporting information.

**Data S2.** Supporting information.

## Data Availability

Research data are not shared.
