# Supplementary figures and images for: Improving Health Outcomes Through Treatment Sequencing Optimization in Multiple Myeloma: A Simulation Model in Transplant‐Ineligible Patients
Source: Cancer Rep (Hoboken). 2024 Oct 7;7(10):e70027. doi: 10.1002/cnr2.70027 (PMC11458883; doi:10.1002/cnr2.70027)

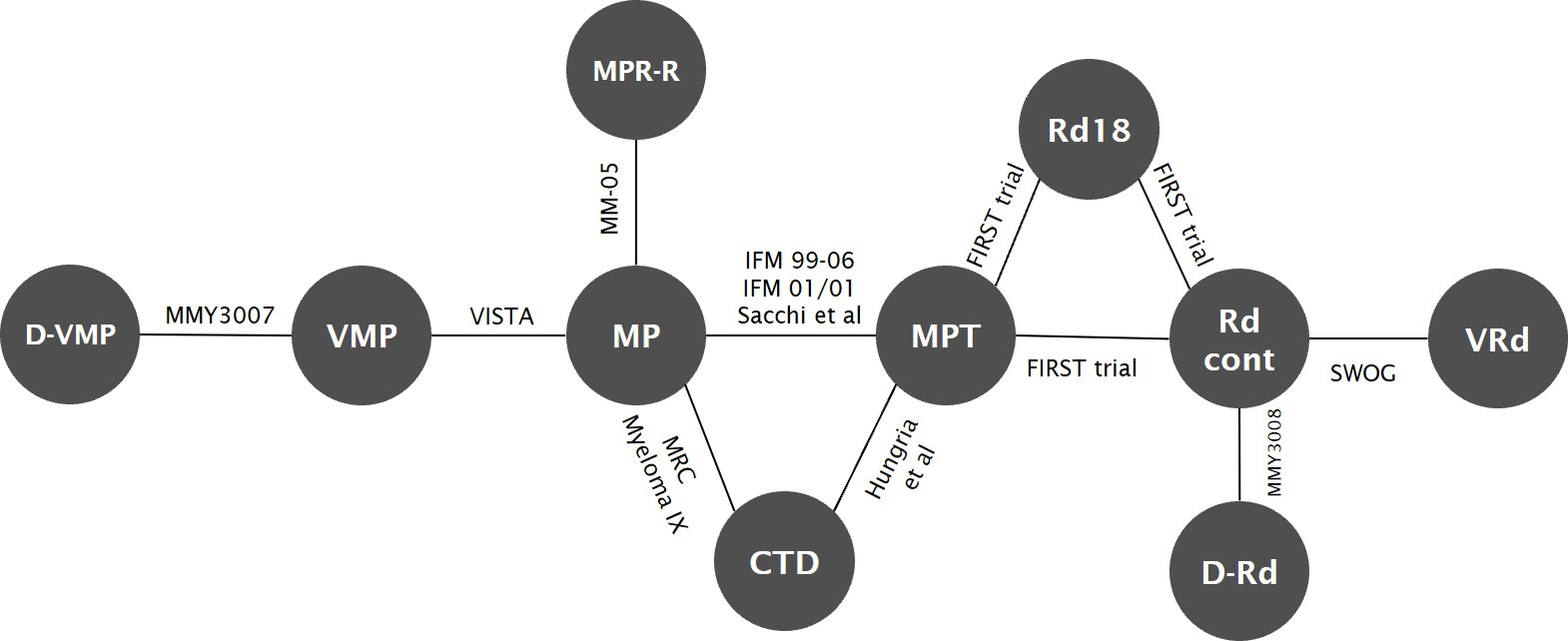

Supplement: Supplementary file 1 — Figure S1. [file CNR2-7-e70027-s003.jpg]

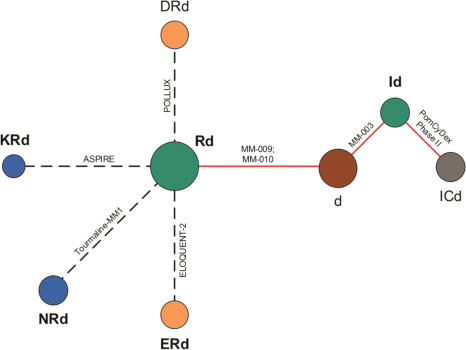

Supplement: Supplementary file 2 — Figure S2. [file CNR2-7-e70027-s002.tif]

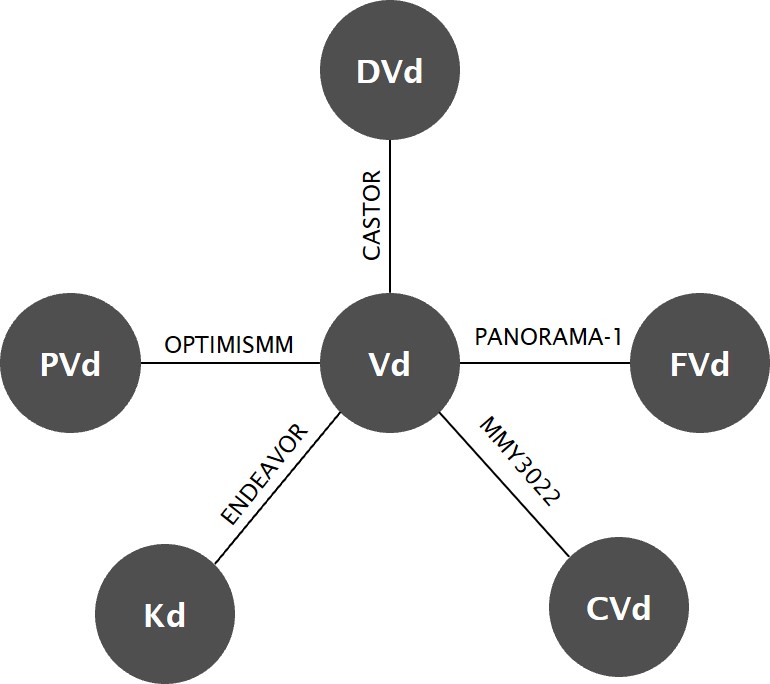

Supplement: Supplementary file 3 — Figure S3. [file CNR2-7-e70027-s001.jpg]
