# Supplementary material for: Improving Health Outcomes Through Treatment Sequencing Optimization in Multiple Myeloma: A Simulation Model in Transplant‐Ineligible Patients
Source: Cancer Rep (Hoboken). 2024 Oct 7;7(10):e70027. doi: 10.1002/cnr2.70027 (PMC11458883; doi:10.1002/cnr2.70027)
Supplement: Supplementary file 4 — Data S1. Supporting information. [file CNR2-7-e70027-s004.docx]

**SUPPLEMENTARY MATERIAL**

**CLINICAL TRIALS**

Clinical trials used to inform the LUMOS model were identified by systematic literature review of peer-reviewed papers and stratified by newly diagnosed, relapsed/refractory and double refractory MM (Table S1).

**Table S1** Clinical trials used in LUMOS model for TIE MM

| First author (year) | Trial name | Title | Intervention | Comparator |
| --- | --- | --- | --- | --- |
| Newly diagnosed TIE MM |  |  |  |  |
| Mateos (2020) | ALCYONE (MMY3007) | Overall survival with daratumumab, bortezomib, melphalan, and prednisone in newly diagnosed multiple myeloma (ALCYONE): a randomised, open-label, phase 3 trial | DVMP | VMP |
| Facon (2019), Bahlis (2019) | MAIA (MMY3008) | Daratumumab + Lenalidomide and Dexamethasone for Untreated Myeloma, Daratumumab + Lenalidomide and Dexamethasone (D-Rd) Versus Lenalidomide and Dexamethasone (Rd) in Patients with Newly Diagnosed Multiple Myeloma (NDMM) Ineligible for Transplant: Updated Analysis of MAIA | DRd | Rd |
| San Miguel (2008) | VISTA | Bortezomib + melphalan and prednisone for initial treatment of multiple myeloma | VMP | MP |
| Palumbo (2012) | MM-015 | Continuous lenalidomide treatment for newly diagnosed multiple myeloma | MPR-R | MP |
| Facon (2007) | IFM 99-06 | Melphalan and prednisone + thalidomide versus melphalan and prednisone alone or reduced-intensity autologous stem cell transplantation in elderly patients with multiple myeloma (IFM 99-06): a randomised trial | MP | MPT |
| Hulin (2009) | IFM 01/01 | Efficacy of melphalan and prednisone + thalidomide in patients older than 75 years with newly diagnosed multiple myeloma: IFM 01/01 trial | MP | MPT |
| Sacchi (2011) | Sacchi et al. | A randomized trial with melphalan and prednisone versus melphalan and prednisone + thalidomide in newly diagnosed multiple myeloma patients not eligible for autologous stem cell transplant | MP | MPT |
| Facon (2018) | FIRST | Final analysis of survival outcomes in the phase 3 FIRST trial of up-front treatment for multiple myeloma | MPT, Rd18, Rd cont. |  |
| Durie (2017), Durie (2018)  Durie (2020) | SWOG S0777 | Bortezomib with lenalidomide and dexamethasone versus lenalidomide and dexamethasone alone in patients with newly diagnosed myeloma without intent for immediate autologous stem-cell transplant (SWOG S0777): a randomised, open-label, phase 3 trial, Longer Term Follow up of the Â Randomized Phase III Trial SWOG S0777: Bortezomib, Lenalidomide and Dexamethasone Vs. Lenalidomide and Dexamethasone in Patients (Pts) with Previously Untreated Multiple Myeloma without an Intent for Immediate Autologous Stem Cell Transplant (ASCT) | Rd cont. | VRd |
| **Relapsed or refractory MM** |  |  |  |  |
| Palumbo (2016) | CASTOR (MMY3004) | Daratumumab, Bortezomib, and Dexamethasone for Multiple Myeloma | DVd | Vd |
| Usmani (2016), Kaufman (2019) | POLLUX (MMY3003) | Efficacy of Daratumumab, Lenalidomide, and Dexamethasone Versus Lenalidomide and Dexamethasone in Relapsed or Refractory Multiple Myeloma Patients with 1 to 3 Prior Lines of Therapy: Updated Analysis of Pollux, Four-Year Follow-up of the Phase 3 Pollux Study of Daratumumab + Lenalidomide and Dexamethasone (D-Rd) Versus Lenalidomide and Dexamethasone (Rd) Alone in Relapsed or Refractory Multiple Myeloma (RRMM) | DRd | Rd |
| Stewart (2015) | ASPIRE | Carfilzomib, lenalidomide, and dexamethasone for relapsed multiple myeloma | KRd | Rd |
| Lonial (2015) | ELOQUENT-2 | Elotuzumab Therapy for Relapsed or Refractory Multiple Myeloma | EloRd | Rd |
| Moreau (2016) | TOURMALINE-MM1 | Oral Ixazomib, Lenalidomide, and Dexamethasone for Multiple Myeloma | Ird | Rd |
| Richardson (2019) | OPTIMISMM | Pomalidomide, bortezomib, and dexamethasone for patients with relapsed or refractory multiple myeloma previously treated with lenalidomide (OPTIMISMM): a randomised, open-label, phase 3 trial | PVd | Vd |
| Dimopoulos (2016) | ENDEAVOR | Carfilzomib and dexamethasone versus bortezomib and dexamethasone for patients with relapsed or refractory multiple myeloma (ENDEAVOR): a randomised, phase 3, open-label, multicentre study | Kd | Vd |
| Kropff (2017) | VCD Phase III | Bortezomib and low-dose dexamethasone with or without continuous low-dose oral cyclophosphamide for primary refractory or relapsed multiple myeloma: a randomized phase III study | VCd | Vd |
| Hjorth (2012) | Nordic Myeloma Study | Thalidomide and dexamethasone vs. bortezomib and dexamethasone for melphalan refractory myeloma: a randomized study | Td | Vd |
| **Double refractory MM** |  |  |  |  |
| Lonial (2016) | SIRIUS (MMY2002) | Daratumumab monotherapy in patients with treatment-refractory multiple myeloma (SIRIUS): an open-label, randomised, phase 2 trial | D | NA |
| Lokhorst (2015) | GEN501 | Targeting CD38 with Daratumumab Monotherapy in Multiple Myeloma | D | NA |
| Richardson PG (2016) | PANORAMA-1 | Panobinostat plus bortezomib and dexamethasone in previously treated multiple myeloma: outcomes by prior treatment. | Vd | FVd |
| Dimopoulos (2018) | ELOQUENT-3 | Elotuzumab + Pomalidomide and Dexamethasone for Multiple Myeloma | EloPd | Pd |
| Attal (2019) | ICARIA-MM | Isatuximab + pomalidomide and low-dose dexamethasone versus pomalidomide and low-dose dexamethasone in patients with relapsed and refractory multiple myeloma (ICARIA-MM): a randomised, multicentre, open-label, phase 3 study | IsaPd | Pd |
| San Miguel (2013) | MM-003 | Pomalidomide + low-dose dexamethasone versus high-dose dexamethasone alone for patients with relapsed and refractory multiple myeloma (MM-003): a randomised, open-label, phase 3 trial | Pd | High dose dexamethasone |
| Kropff (2017) | MMY3022 | Bortezomib and low-dose dexamethasone with or without continuous low-dose oral cyclophosphamide for primary refractory or relapsed multiple myeloma: a randomized phase III study | VCd | Vd |

Abbreviations: DRd, daratumumab + lenalidomide + dexamethasone; DVd, daratumumab + bortezomib + dexamethasone; DVMP, daratumumab + bortezomib + melphalan + prednisone; DVTd, daratumumab + bortezomib + thalidomide + dexamethasone; EloPd, elotuzumab + pomalidomide + dexamethasone; EloRd, elotuzumab + lenalidomide + dexamethasone; IsaPd, isatuximab + pomalidomide + dexamethasone; KRd, carfilzomib + lenalidomide + dexamethasone; MM, multiple myeloma; MP, melphalan + prednisone; MPR, melphalan + prednisone + lenalidomide; MPT, melphalan + prednisone + thalidomide; NA, not applicable; Rd, lenalidomide + dexamethasone; Rd cont., lenalidomide + dexamethasone continuous; Rd18, lenalidomide + dexamethasone for 18 weeks; VCd, bortezomib + cyclophosphamide + dexamethasone; VMP, bortezomib + melphalan + prednisone; VRd, bortezomib + lenalidomide + dexamethasone.

**NETWORK META-ANALYSIS**

**Figure S1** Network meta-analysis to estimate relative efficacy of regimens for newly diagnosed TIE MM patients; circles represent regimens and lines represent head-to-head clinical trials (Table S1); figure was adapted from Facon T et al (14)


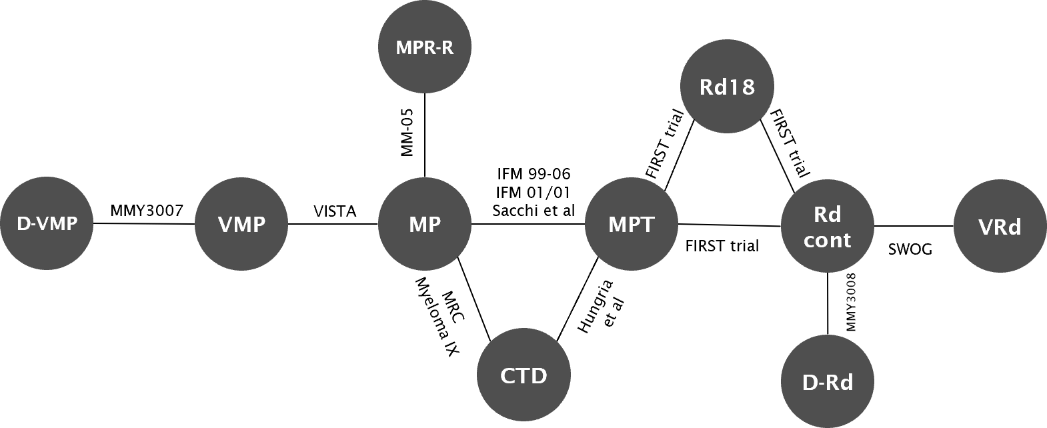


**Abbreviations:** D-Rd, daratumumab + lenalidomide + dexamethasone; D-VMP, daratumumab + bortezomib + melphalan + prednisone; MP, melphalan + prednisone; MPT, melphalan + prednisone + thalidomide; Rd18, lenalidomide + dexamethasone – 18 cycles; Rd cont., lenalidomide + dexamethasone continuous; VMP, bortezomib + melphalan + prednisone; VRd, bortezomib + lenalidomide + dexamethasone.

**Figure S2** Network meta-analysis to estimate relative efficacy of immunomodulatory-containing regimens for relapsed/refractory MM patients; circles represent regimens and lines represent head-to-head clinical trials (Table S1); original figure from Dimopoulos MA et al. (20)


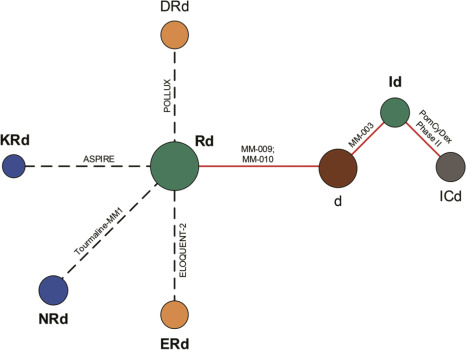


**Abbreviations:** d, dexamethasone; DRd, daratumumab + lenalidomide + dexamethasone; ERd, elotuzumab + lenalidomide + dexamethasone; Id, pomalidomide + dexamethasone; ICd, pomalidomide + cyclophosphamide + dexamethasone; KRd, carfilzomib + lenalidomide + dexamethasone; NRd ixazomib + lenalidomide + dexamethasone; Rd, lenalidomide + dexamethasone.

**Figure S3** Network meta-analysis to estimate relative efficacy of immunomodulatory-free regimens for relapsed/refractory MM patients; circles represent regimens and lines represent head-to-head clinical trials (Table S1); figure was adapted from Zayas J et al. (21)


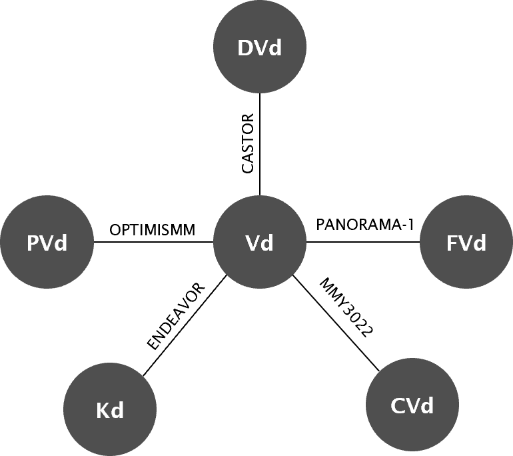


**Abbreviations:** DVd, daratumumab + bortezomib + dexamethasone; EVd, elotuzumab + bortezomib + dexamethasone; CVd, cyclophosphamide + bortezomib + dexamethasone; FVd, panobinostat + bortezomib + dexamethasone; Kd, carfilzomib + dexamethasone; Vd, bortezomib + dexamethasone.

**MODEL VALIDATION**

Quality control checks were conducted on the model at the various stages of its development, to ensure technical model validity including:

1. A manual review of all programming using formulae in Microsoft Excel and code in Visual Basic for Applications
2. Confirmation that mathematical calculations, programming and formulae are consistent with the specification and were logically applied
3. A review of the patient flow sheets
4. A review of the scenario analysis functionality and results
5. Checks for internal validity, comparing model outcomes to the clinical trials
6. Extreme value testing (setting in zero values to parameters and stress testing the model in general) to assess whether the outcomes and changes in outcomes make intuitive sense for the options selected
7. Editorial checks (text descriptions, referencing, navigation and graphical outputs).

Quality control checks were performed by health economists not involved in the development or construction of the model.
